# Phylogenomics and Comparative Genomics Highlight Specific Genetic Features in *Ganoderma* Species

**DOI:** 10.3390/jof8030311

**Published:** 2022-03-18

**Authors:** Yi-Fei Sun, Annie Lebreton, Jia-Hui Xing, Yu-Xuan Fang, Jing Si, Emmanuelle Morin, Shingo Miyauchi, Elodie Drula, Steven Ahrendt, Kelly Cobaugh, Anna Lipzen, Maxim Koriabine, Robert Riley, Annegret Kohler, Kerrie Barry, Bernard Henrissat, Igor V. Grigoriev, Francis M. Martin, Bao-Kai Cui

**Affiliations:** 1 Institute of Microbiology, School of Ecology and Nature Conservation, Beijing Forestry University, Beijing 100083, China; yifeisun2016@163.com (Y.-F.S.); jhxing321@sina.com (J.-H.X.); yuxuanf2000@163.com (Y.-X.F.); jingsi1788@126.com (J.S.); 2 Université de Lorraine, INRAE, UMR Interactions Arbres/Microorganismes (IAM), Centre INRAE Grand Est-Nancy, 54280 Champenoux, France; annie.lebreton@inrae.fr (A.L.); emmanuelle.morin@inrae.fr (E.M.); smiyauchi@mpipz.mpg.de (S.M.); annegret.kohler@inrae.fr (A.K.); 3 Beijing Advanced Innovation Center for Tree Breeding by Molecular Design, Beijing Forestry University, Beijing 100083, China; 4 Max Planck Institute for Plant Breeding Research, Department of Plant Microbe Interactions, 50829 Cologne, Germany; 5 INRAE, Aix Marseille University, UMR1163 Biodiversité et Biotechnologie Fongiques, 13009 Marseille, France; elodie.drula@inrae.fr; 6 U.S. Department of Energy Joint Genome Institute, Lawrence Berkeley National Laboratory, Berkeley, CA 94720, USA; sahrendt0@lbl.gov (S.A.); klcobaugh@lbl.gov (K.C.); alipzen@lbl.gov (A.L.); mkoriabine@lbl.gov (M.K.); rwriley@lbl.gov (R.R.); kwbarry@lbl.gov (K.B.); ivgrigoriev@lbl.gov (I.V.G.); 7 DTU Bioengineering, Technical University of Denmark, 2800 Kongens Lyngby, Denmark; bernard.henrissat@gmail.com; 8 Department of Biological Sciences, King Abdulaziz University, Jeddah 999088, Saudi Arabia; 9 Department of Microbial and Plant Biology, University of California Berkeley, Berkeley, CA 94720, USA

**Keywords:** *Ganoderma*, genomics, secondary metabolism, secretome, terpenes, wood decay

## Abstract

The *Ganoderma* species in Polyporales are ecologically and economically relevant wood decayers used in traditional medicine, but their genomic traits are still poorly documented. In the present study, we carried out a phylogenomic and comparative genomic analyses to better understand the genetic blueprint of this fungal lineage. We investigated seven *Ganoderma* genomes, including three new genomes, *G. australe*, *G. leucocontextum*, and *G. lingzhi*. The size of the newly sequenced genomes ranged from 60.34 to 84.27 Mb and they encoded 15,007 to 20,460 genes. A total of 58 species, including 40 white-rot fungi, 11 brown-rot fungi, four ectomycorrhizal fungi, one endophyte fungus, and two pathogens in Basidiomycota, were used for phylogenomic analyses based on 143 single-copy genes. It confirmed that *Ganoderma* species belong to the core polyporoid clade. Comparing to the other selected species, the genomes of the *Ganoderma* species encoded a larger set of genes involved in terpene metabolism and coding for secreted proteins (CAZymes, lipases, proteases and SSPs). Of note, *G. australe* has the largest genome size with no obvious genome wide duplication, but showed transposable elements (TEs) expansion and the largest set of terpene gene clusters, suggesting a high ability to produce terpenoids for medicinal treatment. *G. australe* also encoded the largest set of proteins containing domains for cytochrome P450s, heterokaryon incompatibility and major facilitator families. Besides, the size of *G. australe* secretome is the largest, including CAZymes (AA9, GH18, A01A), proteases G01, and lipases GGGX, which may enhance the catabolism of cell wall carbohydrates, proteins, and fats during hosts colonization. The current genomic resource will be used to develop further biotechnology and medicinal applications, together with ecological studies of the *Ganoderma* species.

## 1. Introduction

*Ganoderma* species (Ganodermataceae, Polyporales, Basidiomycota) are both ecologically and economically relevant fungi found in forest ecosystems. As wood-decay fungi, *Ganoderma* species efficiently decompose the components of plant cell walls, including lignin, cellulose and hemicellulose [1]. *G. boninense* Pat. causes a basal stem rot (BSR) on oil palm trees [2,3], while *G. lucidum* and *G. australe* have been used for degradation of environmental pollutants [4,5]. As medicines, *Ganoderma* fruiting bodies have been used over the last 2000 years to produce drugs used for improving immunity, and in anti-aging and anti-cancer treatments in humans [6,7,8]. For example, spore powder or basidiocarp slices of *G. lingzhi* Sheng H. Wu, Y. Cao, and Y.C. Dai and *G. tsugae* Murrill are used in Asian traditional medicine to improve health. On the other hand, polysaccharides and glycans extracted from *G. sinense* J.D. Zhao, L.W. Hsu and X.Q. Zhang have been used as clinical drugs [9].

*Ganoderma* is the largest genus in Ganodermataceae including 461 taxa recorded in Index Fungorum (http://www.indexfungorum.org/, accessed on 16 January 2022) as of 15 September 2021. However, only four complete genomes of *Ganoderma* species have been reported at this date. The genome of the well-known medicinal polypore, *G. lucidum*, was published by Chen et al. [10]. This study focused on the analyses of genes encoding cytochrome P450s (CYPs), transporters and regulatory proteins which are related to secondary metabolism (SM) and wood degradation. Then, Binder et al. [11] used the genome of an unknown North American *Ganoderma* species to unravel phylogenetic relationships within the Polyporales. The sequencing and analyses of *G. sinense*, another well-known medicinal fungus, provided new highlights on the genome methylation patterns, small RNA transcriptome, SM and defense processes [12]. Utomo et al. [13] sequenced and analyzed a pathogenic strain of *G. boninense* isolated from an oil palm tree with severe symptoms of BSR disease. Additional genomes are required to explore the full diversity of *Ganoderma* gene sets involved secondary metabolism and wood white-rot decay and identify the genetic traits specific to the different species. Therefore, we sequenced and analyzed three additional genomes of *Ganoderma* species and compared them to other previously sequenced taxa, i.e., *G. australe*, *G. leucocontextum* and *G. lingzhi*.

*Ganoderma australe* is widely distributed on deciduous trees in central, eastern and southern China, and *G. lingzhi* is widely cultivated thanks to its high medicinal value, whereas *G. leucocontextum* was recently described from southwestern China and is now cultivated in Yunnan and Tibet for medicinal production. In addition to these three new genomes, we included in our analysis seven publicly available genomes of *Ganoderma* species. Finally, we compared the *Ganoderma* gene sets to those obtained on 51 Basidiomycota species, including white-rot and brown-rot wood decayers, and biotrophic fungi. Here, we provided new insights on the evolutionary relationships between *Ganoderma* species and other Polyporales, and we identified idiosyncrasies in the gene repertoire involved in SM and secreted enzymes.

## 2. Materials and Methods

### 2.1. Strain and Culture Conditions

Fruiting bodies of *Ganoderma leucocontextum* Dai 12418 was collected on *Quercus*, and the strain was cultured on Potato Dextrose Agar (Potato Extract 200 g, Agar 20 g, Dextrose 20 g, water 1 L); *G. australe* Cui 17254 and *G. lingzhi* Cui 9166 were collected on other angiosperm logs., and the strains were cultured on Malt Extract Agar (Malt Extract 20 g, Agar 18 g, KH_2_PO_4_ 3 g, Glucose 10 g, water 1 L). The strains were cultured for 7–14 days in the dark at 25 °C. The taxonomic affiliation of the three strains was confirmed by phylogenetic analyses based on ITS sequences. Isolates were deposited in the culture collection of the Institute of Microbiology, Beijing Forestry University and are available upon request.

### 2.2. DNA Extractions

For genomic DNA extraction, fresh 7-day-old vegetative agar mycelial cultures grown on cellophane sheets were harvested, snap frozen in liquid nitrogen and stored at −80 °C. High molecular weight genomic DNA of *Ganoderma australe* was extracted by using the sodium dodecylsulfate (SDS) method [14], whereas a modified cetyltrimethyl ammonium bromide (CTAB) method [15] was used for genomic DNA extraction of *G. leucocontextum* and *G. lingzhi*. 

### 2.3. Genome Sequencing and Assembly

The genome of the strain Dai 12418 from *Ganoderma leucocontextum* was sequenced using the Pacific Biosciences platform (PacBio, Menlo Park, CA, USA); 1.5 μg of genomic DNA was sheared to 10 kb using Covaris g-Tube or Diagenode megaruptor tube. The sheared DNA was treated with DNA prep to remove single-stranded ends and DNA damage repair mix followed by end repair/A Tail and ligation of barcoded overhang adapters using SMRTbell Express Template Prep 2.0 Kit (PacBio, Menlo Park, CA, USA). The library was purified with AMPure PB beads and libraries with different barcodes were pooled at equimolar for up to four maximum fungal genomes with a total sum of genome sizes of 200 Mb. A BluePippin size selection (Sage Science, Beverly, MA, USA) was then performed on the pool to remove the shorter peaks physically. PacBio Sequencing primer was then annealed to the SMRTbell template library and sequencing polymerase was bound to them using Sequel II Binding kit 2.0. The prepared SMRTbell template libraries were then sequenced on a Pacific Biosystems’ Sequel II sequencer using 8 M v1 SMRT cells and Version 2.0 sequencing chemistry with 1 × 1800 sequencing movie run times. Filtered subread data was processed to remove artifacts. Mitochondria was assembled separately with the CCS reads using an in-house tool (assemblemito.py), used to filter the CCS reads, and polished with gcpp—algorithm arrow version SMRTLink v8.0.0.80529 (https://www.pacb.com/support/software-downloads, accessed on 16 January 2022). The mitochondria-filtered CCS reads were then assembled with Flye version 2.7.1-b1590 (https://github.com/fenderglass/Flye, accessed on 16 January 2022) (—g 40 M—asm-coverage 50—pacbio-corr) and polished with gcpp—algorithm arrow version SMRTLINK v8.0.0.80529. Contigs less than 1000 bp were excluded.

The *Ganoderma australe* Cui 17254 and *G. lingzhi* Cui 9166 genomes were sequenced using PacBio and Illumina platform. Sequencing libraries for Illumina were generated using NEBNext^®^ Ultra™ DNA Library Prep Kit (NEB, Ipswich, MA, USA). One μg of genomic DNA was fragmented by sonication to 350 bp (400 bp for *G. lingzhi* DNA) using Covaris g-Tube. The DNA fragments were end-polished, A-tailed, and ligated with the full-length adaptor for Illumina sequencing with further PCR amplification. The libraries were purified by AMPure XP system (Beckman Coulter, Brea, IN, USA) and were analyzed for size distribution by Agilent2100 Bioanalyzer and quantified using real-time PCR. The libraries for single-molecule real-time (SMRT) on PacBio platform was constructed with an insert size of 20 kb using the SMRT bell TM Template kit (version 1.0, Pacific Biosciences, Menlo Park, CA, USA). The DNA fragments were repaired DNA damage and ends and prepared blunt ligation reaction. The library was purified with 0.45X AMPure PB beads and size-selection using the BluePippin System (Sage Science, Beverly, MA, USA). The libraries were analyzed for size distribution by Agilent 2100 Bioanalyzer (Agilent, Santa Clara, CA, USA). The whole genome of *G. australe* was sequenced using PacBio Sequel and Illumina NovaSeq PE150 at Beijing Novogene Bioinformatics Technology Co., Ltd. (Beijing, China). The whole genome of *G. lingzhi* was sequenced using PacBio RS II platform and Illumina MiSeq platform at Shanghai Personalbio Technology Co., Ltd. (Shanghai, China). The low-quality reads were filtered (less than 500 bp) to obtain clean data. Preliminary assembly was conducted with SMRTLink v5.0.1 (https://www.pacb.com/support/software-downloads, accessed on 16 January 2022), and long reads (more than 6000 bp) were selected. By the variant Caller module of the SMRT Link software, the arrow algorithm was used to correct and count the variant sites in the preliminary assembly results.

### 2.4. Genome Annotation and Quality Check

The genome of *Ganoderma leucocontextum* was annotated using the JGI Annotation Pipeline with the support of their corresponding Trinity transcriptomes. Both assembly and annotations are available from JGI genome portal in MycoCosm (https://mycocosm.jgi.doe.gov/, accessed on 16 January 2022) [16,17]. The de novo annotation of *G. australe* and *G. lingzhi* were conducted by Augustus v3.03 [18], Genewise v2.4.1 [19] and EvidenceModeler [20].

The quality of the predicted proteomes was evaluated by using the tool Benchmarking Universal Single-Copy Orthologs (BUSCO v.4.1.3) [21] with the Basidiomycota gene set downloaded from https://busco-data.ezlab.org/v4/data/lineages/basidiomycota_odb10.2020-09-10.tar.gz, accessed on 16 January 2022.

### 2.5. Synteny Analyses

The synteny analyses were conducted on the 10 largest scaffolds of each of the selected *Ganoderma* genomes. Pair-wise comparisons and identification of syntenic blocks were performed by using the R package DECIPHER [22] with default parameters. The synteny blocks between every two species were visualized with the R package Circlize [23]. Data management, integration, and visualization were as described in Hage et al. [24].

### 2.6. Phylogenetic Analyses

The phylogenetic analyses of 12 *Ganoderma* species were conducted by Maximum Likelihood (ML) with 23 ITS sequences. *Sanguinoderma* sp. Cui 17238 was used as the outgroup. The ML analyses were performed in RAxML-HPC v. 8.2.3 [25] involving 1000 ML searches under the GTRGAMMA model and 1000 rapid bootstrap replicates with the GTRCAT model to obtain the best tree and ML bootstrap. All trees were viewed in FigTree 1.4.2 (http://tree.bio.ed.ac.uk/software/figtree/, accessed on 16 January 2022). The ML bootstrap ≥ 50% were presented on topologies from ML analyses.

### 2.7. Phylogenomic Analyses

A total of 58 Basidiomycota species was used for the phylogenomic analyses, including 40 white-rot fungi, 11 brown-rot fungi, four ectomycorrhizal fungi, a single endophyte and two pathogens. *Melampsora larici-populina* Kleb. and *Ustilago maydis* (DC.) Corda were used as outgroup species (Table 1).

The orthologous protein clusters of the 58 proteomes were identified with OrthoFinder v2.4.0 [51]. The sequences of each of 143 single copy, conserved orthologs were aligned using MAFFT v7.471 [52]. Poorly aligned regions were removed with Trimal v1.4.1 [53]. Based on these alignments, a maximum likelihood phylogenomic tree was constructed by RAxML-NG v0.9.0 [54] using partitions corresponding to an orthologous group, and their associated best-fit model for each partition of the concatenate protein alignments were estimated by Modeltest-NG v0.1.6 (Table S1) [55].

### 2.8. Annotation of Transposable Elements

Transposable elements (TEs) were identified as described in Payen et al. [56]. Briefly, de novo repeat sequences were predicted in unmasked genome assemblies of 58 genomes, using RepeatScout 1.0.6 [57]. Sequences ≥ 100 bp and ≥10 occurrences were filtered out. The selected sequences were annotated by searching homologous sequences against the fungal references in REPBASE v.22.08 (http://www.girinst.org/server/RepBase/index.php, accessed on 16 January 2022) using tBLASTx [58]. The coverage of TEs in the genomes, including unknown TEs, was estimated by REPEATMASKER open 4.1.1 (http://www.repeatmasker.org, accessed on 16 January 2022).

### 2.9. Protein Functionnal Annotation

The gene clusters related to SM biosynthetic pathways were predicted by antiSMASH 4.2.0 [59] and visualized along with the species phylogenetic relationship on iTOLv5 (https://itol.embl.de/, accessed on 16 January 2022) [60]. PFAM domain searches were performed with HMMER [61]. The most abundant PFAM protein domains (abundance > 100) were visualized along with the species phylogenetic relationship on iTOLv5. Secreted proteins were predicted as described in Pellegrin et al. [62], and proteins with a size < 300 amino acids were identified as small secreted proteins (SSP). Carbohydrate-active enzymes (CAZymes) were manually curated by the CAZy team (http://www.cazy.org, accessed on 16 January 2022). The annotation of secreted proteases and lipases was performed by BLASTP search (E-value = 10^−5^) against MEROPS (http://merops.sanger.ac.uk/, accessed on 16 January 2022) and Lipase Engineering Database (http://www.led.uni-stuttgart.de/, accessed on 16 January 2022).

## 3. Results

### 3.1. Main Genome Features

Three *Ganoderma* species were newly sequenced in this study: *Ganoderma australe* strain Cui 17254, *G. leucocontextum* strain Dai 12418 and *G. lingzhi* strain Cui 9166. The size of the *G. australe* assembly was 84.27 Mb and 20,460 protein-coding genes were predicted (Table 2). This is the largest *Ganoderma* genome sequenced to date. The size of the genome assemblies for *G. leucocontextum* and *G. lingzhi* was lower at 60.34 and 60.56 Mb, and 15,007 and 16,592 protein-coding genes were predicted on the assemblies, respectively. Between 73.1% to 99.8% of a benchmark set of conserved fungal genes (BUSCO) were found in genome assemblies, indicating that assembled genomes captured most of the coding gene space, although the gene annotation for *G. lingzhi* appeared to be more fragmented (Table 2).

**Figure 1:**
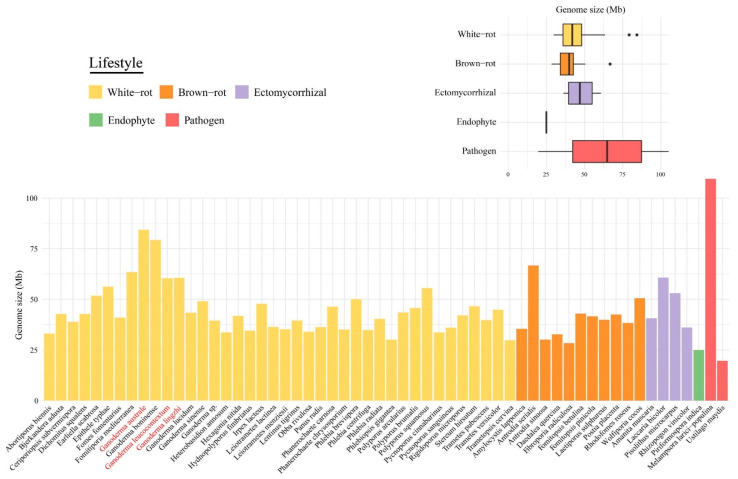
Comparison of the genome size among the 58 selected species. Bars represent size of 58 genomes and boxplots show proportion of genome size in different lifestyles. The new genomes are shown in red.

We compared the three newly sequenced *Ganoderma* genomes to four published *Ganoderma* genomes and 51 other Basidiomycota genomes. The genomes size of the 58 investigated species ranged from 19.66 to 109.88 Mb (Figure 1), with 6785 to 26,226 predicted genes (Table S2). Except for *Melampsora larici-populina*, *Ganoderma* species displayed the largest genomes, i.e., *G. australe* and *G. boninense* were two-to three-fold larger than other wood decayers. No significant differences (*p* > 0.05) in the average genome size were found between white-rot fungi, brown-rot fungi and ectomycorrhizal fungi. The number of duplicated BUSCO genes was higher (23.4%) in *G. australe*. The synteny analyses showed no evidence for whole-genome or segmental duplications (Figure S1), it may indicate a polymorphic dikaryon (Table S2).

### 3.2. Macrosynteny between Ganoderma Genomes

The top 10 scaffolds of each *Ganoderma* genomes, covering 13% to 75% of the whole assemblies, were selected to perform a macrosynteny analysis (Figure S2, Table S3A). We observed the highest percentage of syntenic segments (72%) between *G. lingzhi* and *G. lucidum*, while *G. boninense* and *G. leucocontextum* showed a lower rate when compared to other species (Table S3B), reflecting a higher genome divergence.

### 3.3. Phylogenetic Analyses of Ganoderma Strains

**Figure 2:**
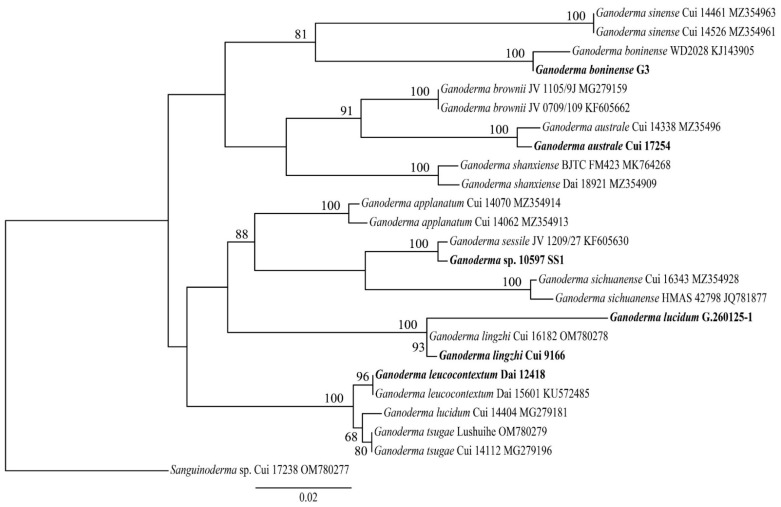
ML analyses of 12 *Ganoderma* strains based on ITS sequences. ML bootstrap values higher than 50% are shown. Stains with sequenced genomes are shown in bold.

The phylogenetic relationship of 12 *Ganoderma* species was conducted based on 23 ITS sequences and one *Sanguinoderma* sp. as outgroup (Figure 2). Two *G. lucidum* strains were not clustered together indicating that the identification of *G. lucidum* G.260125-1 should be considered as *G. lingzhi* actually. *Ganoderma* sp. 10597 SS1 clustered with *G. sessile* suggesting that this strain may pertain to this sessile species in this phylogeny.

### 3.4. Phylogenomic Analyses of the Ganoderma Species and Related Polyporales

**Figure 3:**
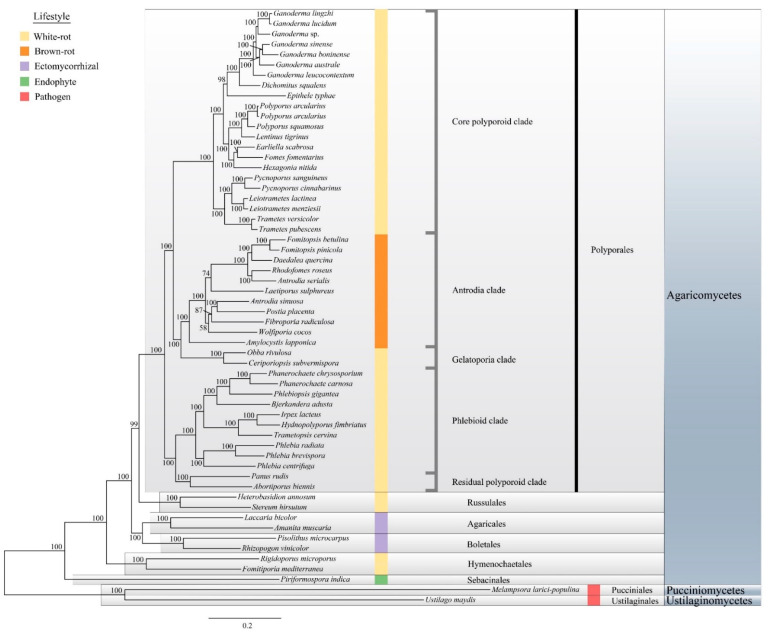
Phylogenetic relationship within the *Ganoderma* clade and 58 selected Basidiomycota species. The ‘best tree’ resulting from maximum likelihood (ML) analyses based on 143 single-copy genes and ML bootstrap values are shown. Colors are coded for the five lifestyles.

Our phylogenetic analyses, based on 143 single-copy conserved protein sequences, was in agreement with previous phylogenetic analyses using single or multi-locus approaches [24,63]. We also identified five major clades in Polyporales: the core polyporoid clade, the antrodia clade, the gelatoporia clade, the phlebioid clade, and the residual polyporoid clade. *Ganoderma* species clustered in the core polyporoid clade with a high bootstrap value (Figure 3, 100% ML bootstrap). The phylogenomic status of *Ganoderma* species was consistent with the macro-synteny conservation results.

The taxonomic status of *Rigidoporus microporus* has been changed to Hymenochaetales [64], and here, it was confirmed again by phylogenomic analyses. Among the sampled species, the brown-rot fungi and the ectomycorrhizal fungi formed monophyletic groups. The present analyses also confirmed that the white-rot lifestyle is evolutionary polyphyletic.

### 3.5. Transposable Element Identification

**Figure 4:**
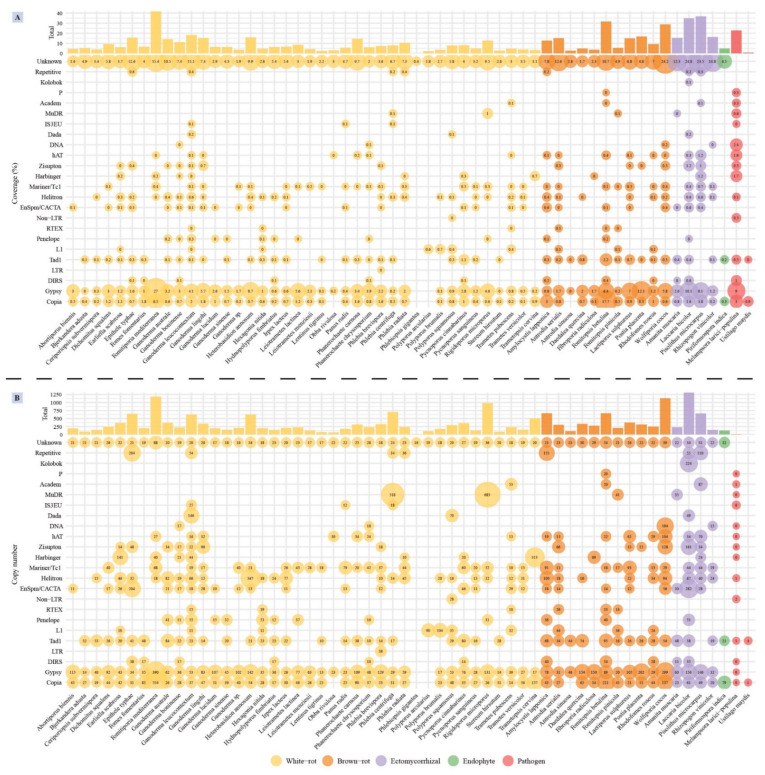
(**A**) Distribution and coverage of transposable elements (TEs) identified in the 58 selected genomes. The bubble size is proportional to the coverage of each of transposable elements (shown inside the bubbles). The bars on top show the total coverage per genome. (**B**) The copy number of transposable elements (TEs) identified in the 58 genomes. The bubble size is proportional to TE copy number (shown inside the bubbles). The bars on top show the total copy number per genome. Color codes for the five fungal lifestyles are shown at the bottom of the figure.

The dominant TEs in *Ganoderma* species belonged to the *Gypsy* and *Copia* families of long terminal repeats (LTR) retrotransposons. Besides, the proportion of unknown TEs in *Ganoderma* species was large, especially in *G. australe* (10.5% of the total assembly) and *G. lingzhi* (11.1% of the total assembly), indicating they likely played a key role in genome rearrangements. *G. leucocontextum* displayed the largest TE coverage (18.32% of the total assembly), and the most diverse TE distribution, including simple repeats (*Repetitive*), *IS3EU* and *Dada* of DNA transposon, *RTEX* and *L1* of non-LTR retrotransposon which are unique repeat elements in *Ganoderma* species (Figure 4A). The number of *IS3EU* and *Dada* sequences in *G. leucocontextum* was significantly larger comparing with other species (Figure 4B). Although occurring at moderate copy numbers, the *G. boninense* genome contained unique TE families, such as the *DIRS* LTR retrotransposons and several DNA repeated elements. *G. australe* also contained more copy of *Tad1* non-LTR retrotransposon and *Helitron* DNA transposons by comparing to other *Ganoderma* species.

TE coverage in the 58 analyzed genomes ranged from 0.37% (*Phlebiopsis gigantea*) to 41.69% (*Fomitiporia mediterranea*) (Table S4). Comparing to other lifestyle fungi, white-rot fungi have low TE coverage except *F. mediterranea* (*p* < 0.01), among which *Ganoderma* species have relatively higher TE coverage (*p* < 0.05). Ectomycorrhizal fungi showed the larger repeat element coverage, in which unknown TE elements were the most abundant (>12%).

### 3.6. Biosynthetic Gene Clusters

**Figure 5:**
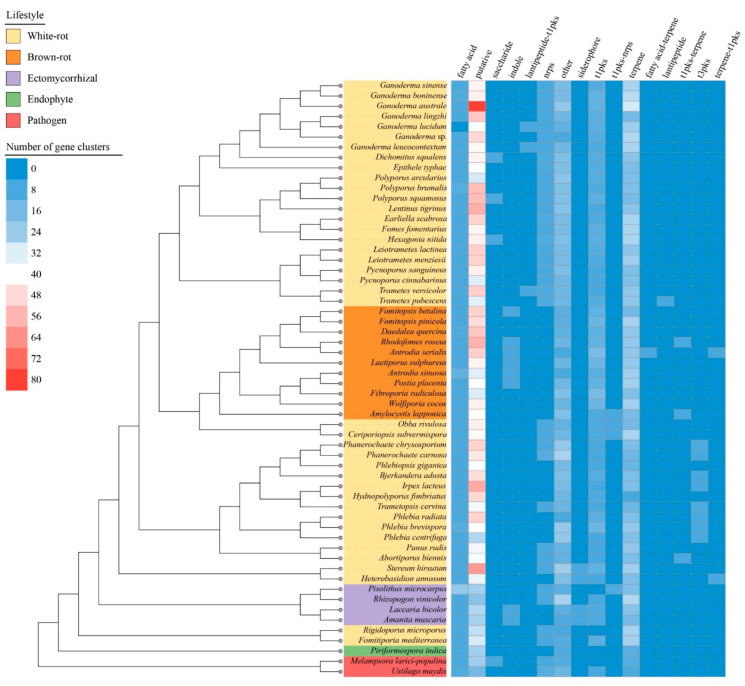
Presence and abundance of the gene clusters involved in secondary metabolite biosynthesis along with the species phylogenetic relationship between the 58 selected fungal species. The heatmap depicts the number of the gene clusters according to the color scale from blue to red. Color codes for the five fungal lifestyles are shown at the top left of the figure.

We identified a total of 16 types of SM biosynthetic clusters (Figure 5, Table S5). Except for the *Rhizopogon vinicolor*, the pathogens, ectomycorrhizal fungi and endophytes generally had a lower number of genes involved in SM than saprotrophic species. This difference was mainly driven by a lower content in t1pks, terpenes related genes and miscellaneous genes tagged as “putative” and “other” by the antiSMASH software. The antrodia clade composed of brown-rot species was segregated from other Polyporales clades by an enrichment in t1pks, indole, and fatty acid associated genes, and a depletion in NRPS. Among white-rot fungi in Polyporales, the phlebioid clade displayed a higher content in t3pks and a lower content in terpenes associated genes. The core polyporoid clade contained the species with diverse sets of biosynthetic genes clusters. Gene clusters involved in terpene synthesis and unknown metabolites (i.e., putative SM gene clusters) were enriched in *Ganoderma* species, especially in *G. australe* which contained 31 terpene associated gene clusters and 80 putative biosynthetic gene clusters. Noteworthy, *G. lingzhi* and *G. lucidum* as the main medically relevant species showed the lowest number of terpenes associated gene clusters, whereas *G. australe* and the pathogenic *G. boninense* had the highest content. *G. lucidum*, *G. leucocontextum*, *Trametes versicolor*, and *T. pubescens* genomes encoded a cluster related to lantipeptide production, and the first three were identified as associated with a t1pks.

### 3.7. Pfam Protein Domains Found in the Genomes

**Figure 6:**
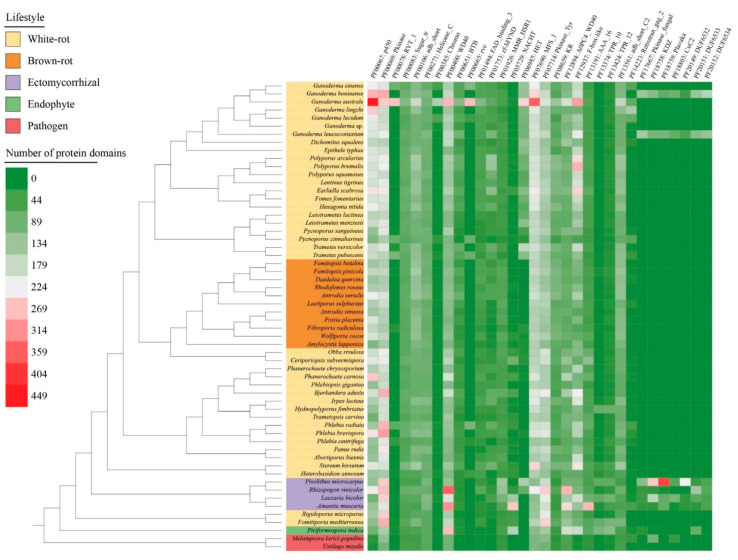
Distribution and abundance of the top 100 Pfam protein domains in the selected 58 species. The heatmap depicts the protein domain copy number according to the color scale from purple to green. Color codes for the five fungal lifestyles are shown at the top left of the figure.

More than 5000 Pfam protein domains were identified in the 58 selected genomes. A total of 32 Pfam categories were sorted according to their gene copy number (>100) (Figure 6, Table S6). *Ganoderma australe* showed the higher number of Pfam protein domains, i.e., five-fold larger than *Ustilago maydis* (with 864 protein domains). In *G. australe*, 13 protein domains were prominent, including cytochrome P450s (PF00067: p450) involved in SM, heterokaryon incompatibility protein (PF06985: HET) often related to vegetative incompatibility (VI) and membrane transporters of the major facilitator superfamily (PF07690: MFS_1). Besides, other protein domains putatively playing a role in epigenetic regulation (PF00078: RVT_1, PF00385: Chromo, PF00665: rve) and protein–protein interactions (PF12937: F-box-like) were also enriched in *G. australe*. Comparing to other *Ganoderma* species, *G. boninense* and *G. leucocontextum* encoded additional protein domains (e.g., PF17667: Pkinase_fungal, PF18758: KDZ, PF18759: Plavaka, PF18803: CxC2, PF20149: DUF6532, PF20151: DUF6533, and PF20152: DUF6534), which were only found in parasitic and symbiotic fungi. Protein kinases domains (PF00069: Pkinase, PF07714: Pkinase_Tyr) and protein–protein interactions domains (PF00400: WD40, PF12894: A0PC4_WD40) were especially enriched in ectomycorrhizal fungi.

### 3.8. The Predicted Secretome

**Figure 7:**
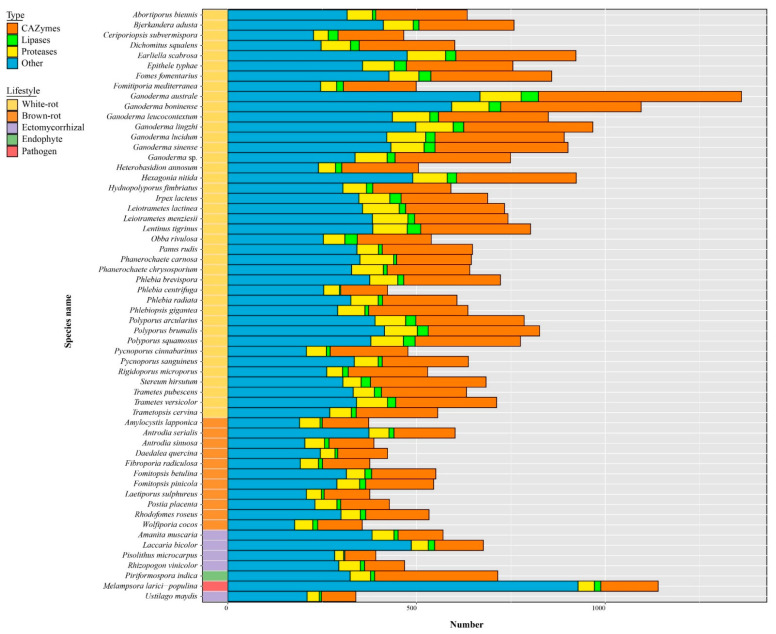
Distribution of predicted secreted proteins (i.e., secretome) in *Ganoderma* species and other selected fungi. Bars represent the gene copy number for CAZymes, lipases, proteases, and SSPs. Color codes for the five fungal lifestyles are shown at the top left of the figure.

**Figure 8:**
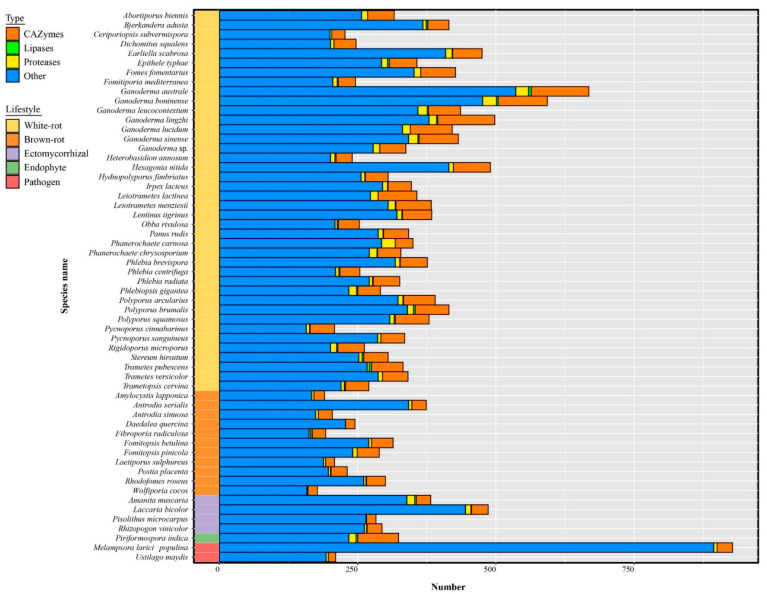
Distribution of small secreted proteins (SSPs) in *Ganoderma* species and other selected fungi. Bars represent the number of annotated (CAZymes, lipases, proteases) and other SSPs. All species are annotated by five lifestyles.

*Ganoderma* species have the largest secretome among the 58 selected fungal species (Figure 7, Table S7). Small secreted proteins (SSPs) represented 41% to 81% of the total secreted proteins and 4% to 28% SSPs were annotated as CAZymes, lipases, or proteases. Most known SSPs were annotated as CAZymes, especially enriched in *G. australe* and *G. lingzhi* (Figure 8, Table S8).

**Figure 9:**
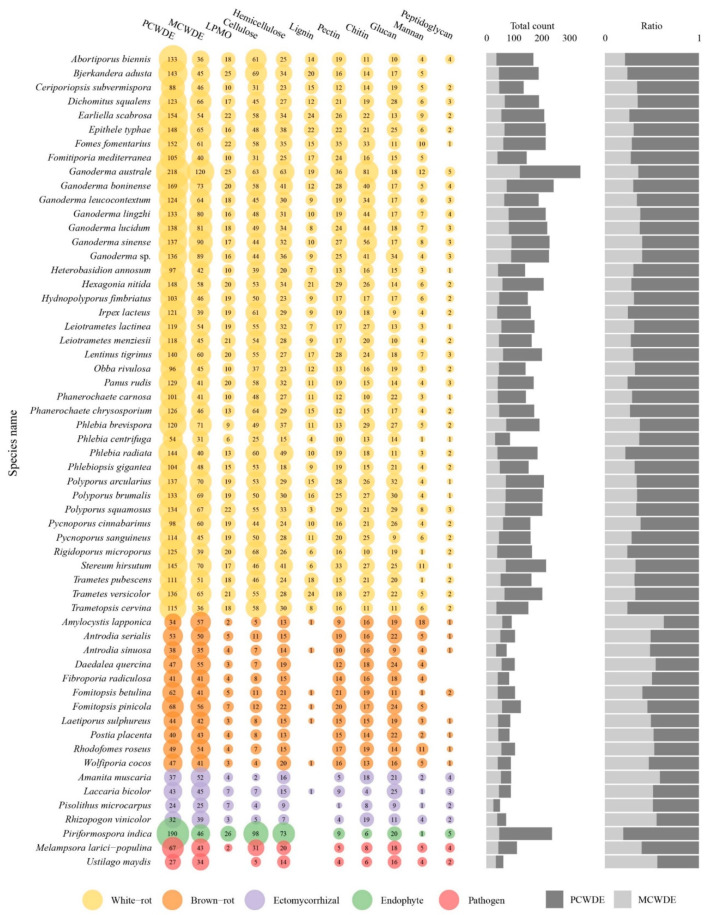
Distribution of secreted CAZymes involved in plant and microbial cell wall degradation in *Ganoderma* species and other selected fungal genomes. The bubble size is proportional to the number of secreted CAZymes grouped for 11 categories. Colors are coded by five lifestyles. The bar plots show the count of genes involved in PCWDE and MCWDE (**left**), and the ratio of PCWDE to MCWDE (**right**).

As secreted CAZymes plays a key role in wood degradation, the evolution of their content through Polyporales was investigated. The different clades in Polyporales have a distinctive CAZyme content and the *Ganoderma* species also contained a distinctive CAZyme repertoire within the core polyporoid clade. The antrodia clade composed of brown-rot fungi have a two-fold lower content of secreted CAZymes compared to the white-rot species in Polyporales (141 ± 24 vs. 237 ± 71 respectively, Table S9). Among the 148 CAZymes sub-families (grouped in 101 families) with secreted genes representatives, 48 (grouped in 38 families) exhibited a lower number of genes in brown-rots compared to white-rots in Polyporales (BM test, FDR padj < 0.01). It included cellulolytic enzymes (GH6, GH7), LPMOs (AA9), ligninolytic PODs (AA1_1), heme-associated PODs (AA2), the carbohydrate-binding module CBM1, CAZymes involved in bacterial cell wall degradation (GH25, GH79), fungal cell wall degradation (GH20, GH76, GH92, GH128, GH135, GH152), and other CAZymes involved in plant cell wall degradation. CAZymes was globally enriched in the core polyporoid clade compared to the phlebioid clade. This enrichment was associated to the expansion of AA1, AA14, CE1, EXPN, GH16, GH17, GH18, GH25, and GH30 families. Of note, the AA7 family was absent from the phlebioid clade and AA12 was absent from the core polyporoid clade. Among the core polyporoid clade, the *Ganoderma* species have enriched CAZymes, and 11 families were expanded, including GH18, GH16, AA1, GH43, CE16, GH3, GH128, GH47, GH115, GH25, and GH1 families. Whereas only PL4 was enriched (and only found) in the other species of the core polyporoid clade. Compared to other species, *G. australe* had the largest set of secreted CAZymes, in which GH18 and EXPN families were highly enriched in this species. It also contained the largest repertoire of PCWDEs, MCWDEs, and enzymes acting on pectin, peptidoglycans, and chitin (Figure 9, Table S10).

A total of 3880 genes encoding for secreted proteases (in 73 MEROPS families) were identified in 58 fungal genomes (Table S11). Eight protease families (A01A, G01, M28E, M36, S08A, S10, S28, and S53) have more than 100 members, and the largest represented protease subfamily was A01A with 1332 proteins. No significant differences were found between saprotrophic and symbiotic species (*p* > 0.05), but five protease families (C40, M24X, M57, M77, S08B) were only found in white-rot fungi, although with low gene copy numbers. Among these families, the M57 protease family including four proteins, was found in *Ganoderma australe*, *G. leucocontextum*, *G. lingzhi,* and *G. lucidum*. In addition, *Ganoderma* species have more G01, M28E, and M35 proteases.

Comparing to other lifestyles, white-rot fungi have the higher set of secreted lipases (*p* < 0.001) and among the 58 analyzed fungal secretomes, *Ganoderma australe* contained the highest number of secreted lipases, the most abundant being GGGX lipases (Table S12). Most genomes displayed less than seven GX lipases, except for *G. boninense* and *G. leucocontextum* with ten and nine GX lipases respectively. They are prominent enzymes catalyzing a wide range of reactions on various cellular substrates [65,66].

## 4. Discussion

In this study, we provided three newly sequenced genomes of ecologically and economically relevant *Ganoderma* species. A phylogeny, based on 23 rDNA ITS sequences from 12 *Ganoderma* species, allowed us to determine the phylogenetic status of the newly sequenced species in *Ganoderma*. This phylogeny concurred with previous studies which were carried out by using a smaller set of *Ganoderma* species [11,63,67], except for *G. lucidum*. Indeed, this species displayed inconsistence between the two available strains, suggesting a misidentification of *G. lucidum* G.260125-1 strain which was purchased from a company. According to our study, this strain may pertain to the *G. lingzhi* which is widely cultivated for its medicinal usage in China [7]. This phylogeny also indicated that *Ganoderma* sp. 10597-SS1 probably pertained to *G. sessile*. The phylogenomic analyses based on 58 genomes, including seven *Ganoderma* species, supported the ITS phylogenetic result and confirmed that *Ganoderma* belong to the core polyporoid clade of Polyporales as defined by Justo et al. [63].

*Ganoderma* species have been used for centuries in traditional medicine thanks to their well-known arsenal of antimicrobial, anti-aging, antioxidant, anti-inflammatory, and immunomodulating compounds (e.g., polysaccharides, triterpenoids, and peptides) [68,69]. The drastic reduction of sequencing cost in the last decades allowed genome-wide mining of medicinal compound. As a result, the number of new genomes rapidly increased over the year, such as genomes of *G. tsugae* CCMJ4178 [67] and *G. leucocontextum* DH-8 [70]. The strain DH-8 of *G. leucocontextum* was newly sequenced and its genome was 50.05 Mb with 58 scaffolds [70], the genome of strain Dai 12418 of *G. leucocontextum* sequenced in our study was 60.34 Mb with 843 scaffolds. In our study, 21 terpene gene clusters were predicted in strain Dai 12418 against the 10 terpene gene clusters predicted in strain DH-8. The two-fold enrichment in strain Dai 12418 compared to strain DH-8 was unexpected, despite taking into differences in annotation tools and the potential bias of assembly quality. It may suggest a substantial intraspecific genome polymorphism. Direct comparisons of other functional gene categories were hampered by major differences in annotation methodologies. For example, Liu et al. [70] predicted 614 CAZymes genes using HMM, while we only found 291 CAZymes genes, using the in-house pipeline from the CAZymes database followed by expert manual curation.

One of the main targets for medically-relevant products are the secondary metabolites associated genes, especially the terpenes in *Ganoderma* species. Compared to other species, the terpene genes of *Ganoderma* species were indeed expanded, however, substantial differences were observed among this genus. *G. lingzhi* and *G. lucidum* as the main medically species have the lowest number of terpenes associated clusters whereas the widespread *G. australe* and the pathogenic *G. boninense* have the highest content. Terpene related gene expansion in *G. australe* and *G. boninense* might explain their ecological ability to develop on a broader set of substrates. On the other hand, a shift of the terpenome composition in *G. lingzhi*, *G. tsugae*, and *G. leucocontextum* might have led to the production of terpenes with beneficial properties for human health. This study also allowed us to identify a polyketide synthase complex involved in the synthesis of the antibacterial lantipeptide [71] in *G. lucidum*, *G. leucocontextum*, *Trametes versicolor*, and *T. pubescens*, likely explaining the use of formulations based on these fungi as antibiotics [10,72,73,74]. Besides, the large content of gene clusters encoding unknown biochemical function(s) were found in Polyporales species, especially *G. australe*, suggested an outstanding ability to synthesize a large set of secondary metabolites of yet unknown function. This confirmed that the economically and medically relevant secondary metabolites of these fungi represent an untapped resource.

As Polyporales is an important group of wood decayers, we investigated the decay ability of these species by analyzing their secretome. The current evolution was posited that early Agaricomycetes were saprotrophic and different lifestyles were derived from it [75,76]. It also supported that the white-rot lifestyle is linked to the ability to degrade lignin. The acquisition of lignin degradation in the Agaricomycetes was estimated in the late Carboniferous and further evolved with the evolution of lignin complexity in Polyporales and Agaricales taxa [77]. The monophyletic antrodia clade, only composed of brow-rot species, was derived from white-rot lineages in current phylogenomic analyses. According to Baldrian and Valášková [75], brown-rot fungi, despite having several independent origins, were associated with the loss of ligninolytic PODs, heme dye-decolorizing PODs, heme-thiolate POD/peroxygenases (HTPs), cellulolytic enzymes (GH6, GH7, LPMO), and the carbohydrate-binding module CBM1 reduced their ability to degrade lignin into its partial modification by releasing Fenton-generated hydroxyl radicals in the colonized material. This statement was confirmed in the antrodia clade, in which those losses were also accompanied by losses in other PCW-degrading enzymes, and FCW- and BCW-degrading enzymes. The enhanced ability of white-rot fungi to decompose substrates was also marked by a secreted lipase enrichment. White-rots specific proteases (C40, M24X, M57, M77, S08B) were identified, and they were related to genetic regulation, bacterial cell-wall modification, nutrient transformation, and other cellular physiological functions.

The various white-rot Polyporales clades also displayed divergent wood degrading abilities. The potential ligninolytic ability and more generally PCW degradation ability of the core polyporoid clade was the highest among Polyporales due to the expansion of ligninolytic PODs (AA1) and hemicellulose degrading enzyme (AA14, CE1, GH30). The difference among white-rot clades also showed in the enzyme arsenal to compete with other microbes for substrates and copy with biotic threats. The specific expansion in BCW-degrading enzyme (GH25) and FCW-degrading enzyme (GH16, GH17, GH18) was also observed in the core polyporoid clade. *Ganoderma* species deepened their lignocellulose degradation ability with further expansion of the ligninolytic PODs (AA1), hemicellulose (GH43, CE16, GH115), BCW- (GH25), and FCW- (GH16, GH18, GH128, GH47) degrading enzymes. Aminopeptidase Ap1 (M28E), deuterolysin (M35) and scytalidoglutamic peptidase (G01) were also enriched in *Ganoderma* species, but further functional analyses are needed to clarify their role(s). Noteworthy, *G. australe* showed an even wider degrading gene repertoire including higher PCW-degrading CAZyme, lipase and protease content. Similarly, numerous cytochrome P450 monooxygenases (PF00067: p450) were found in *G. australe*. These enzymes are known for their role in lignin and xenobiotic degradation [78]. This pattern supported the known ability of *G. australe* to more efficiently decompose lignocellulose than other *Ganoderma* species. An increased CAZyme repertoire in BCW- and FCW-degrading enzymes was also found in *G. australe*, which could perform the higher competitiveness than other microorganism for the substrate during colonization on the hosts. Associated with its enriched number of biosynthetic genes and clusters, such as lytic transglycolase (PF03330) and cerato-platanins (PF07249), this repertoire could be used to combat with miscellaneous biotic threats.

The abundance of HET genes related to heterokaryon incompatibility, MFS transporters, and epigenetic regulation in *Ganoderma australe* indicated that these mechanisms play a key role in the stable reproduction and evolution of this wood decayer [79,80,81]. With 168 protein domains detected among seven *Ganoderma* species (PF00249, PF08914, PF11831, PF12776, PF13837, PF13921, PF15963), MYB transcription factors were also abundant in *Ganoderma* species. These transcription factors are the largest transcription factor families in eukaryotic organisms and play key role in variable development and physiological activities [82,83]. Wang et al. identified 75 MYB transcription factors in five *Ganoderma* species after manually curation, and of the gene copy number found in each species was lower than the detected results in our study. This is likely due to the differences between strains and technical methods used here. Further exploration of MYB genes can help to clarify its potential function during the growth and development of *Ganoderma* fungi.

The present comparative analysis of the publicly available *Ganoderma* genomes revealed a series of genetic features specific to this lineage of wood decayers. This study provided foundational information to characterize further ecological traits of this important group of decomposers. In addition, this information will be used to characterize the regulation of genes involved in SM biosynthesis pathways at the transcriptomic level, including antimicrobial compounds and medicinal drugs.

## Figures and Tables

**Table 1:** List of the 58 genomes used for phylogenomic and comparative analyses. The new genomes are shown in bold.

Species Name	Strain	Reference	Lifestyle	Order	Clade
*Abortiporus biennis*	CIRM-BRFM1778	[24]	White-rot	Polyporales	residual polyporoid clade
*Bjerkandera adusta*	HHB-12826-SP	[11]	White-rot	Polyporales	phlebioid clade
*Ceriporiopsis subvermispora*	B	[26]	White-rot	Polyporales	gelatoporia clade
*Dichomitus squalens*	LYAD-421 SS1	[27]	White-rot	Polyporales	core polyporoid clade
*Earliella scabrosa*	CIRM-BRFM 1817	[24]	White-rot	Polyporales	core polyporoid clade
*Epithele typhae*	CBS 203.58	[24]	White-rot	Polyporales	core polyporoid clade
*Fomes fomentarius*	CIRM-BRFM 1821	[24]	White-rot	Polyporales	core polyporoid clade
*Fomitiporia mediterranea*	MF3/22 #7	[27]	White-rot	Hymenochaetales	-
***Ganoderma australe***	**Cui 17254**	**this study**	**White-rot**	**Polyporales**	**core polyporoid clade**
*Ganoderma boninense*	G3	[13]	White-rot	Polyporales	core polyporoid clade
***Ganoderma leucocontextum***	**Dai 12418**	**this study**	**White-rot**	**Polyporales**	**core polyporoid clade**
***Ganoderma lingzhi***	**Cui 9166**	**this study**	**White-rot**	**Polyporales**	**core polyporoid clade**
*Ganoderma lucidum*	G.260125-1	[10]	White-rot	Polyporales	core polyporoid clade
*Ganoderma sinense*	ZZ0214-1	[12]	White-rot	Polyporales	core polyporoid clade
*Ganoderma* sp.	10597 SS1	[11]	White-rot	Polyporales	core polyporoid clade
*Heterobasidion annosum*	TC32-1	[28]	White-rot	Russulales	-
*Hexagonia nitida*	CIRM-BRFM 1802	[24]	White-rot	Polyporales	core polyporoid clade
*Hydnopolyporus fimbriatus*	CBS384.51	[24]	White-rot	Polyporales	phlebioid clade
*Irpex lacteus*	CCBAS Fr. 238 617/93	[24]	White-rot	Polyporales	phlebioid clade
*Leiotrametes lactinea*	CIRM-BRFM 1664	[24]	White-rot	Polyporales	core polyporoid clade
*Leiotrametes menziesii*	CIRM-BRFM 1781	[24]	White-rot	Polyporales	core polyporoid clade
*Lentinus tigrinus*	ALCF2SS1-7	[29]	White-rot	Polyporales	core polyporoid clade
*Obba rivulosa*	3A-2	[30]	White-rot	Polyporales	gelatoporia clade
*Panus rudis*	PR-1116 ss-1	[24]	White-rot	Polyporales	residual polyporoid clade
*Phanerochaete carnosa*	HHB-10118-Sp	[31]	White-rot	Polyporales	phlebioid clade
*Phanerochaete chrysosporium*	RP-78	[32]	White-rot	Polyporales	phlebioid clade
*Phlebia brevispora*	HHB-7030 SS6	[11]	White-rot	Polyporales	phlebioid clade
*Phlebia centrifuga*	FBCC195	[33]	White-rot	Polyporales	phlebioid clade
*Phlebia radiata*	FBCC0043-79	[34]	White-rot	Polyporales	phlebioid clade
*Phlebiopsis gigantea*	5-6	[35]	White-rot	Polyporales	phlebioid clade
*Polyporus arcularius*	HBB13444	[36]	White-rot	Polyporales	core polyporoid clade
*Polyporus brumalis*	BRFM 1820	[37]	White-rot	Polyporales	core polyporoid clade
*Polyporus squamosus*	CCBS 676	[24]	White-rot	Polyporales	core polyporoid clade
*Pycnoporus cinnabarinus*	BRFM 137	[38]	White-rot	Polyporales	core polyporoid clade
*Pycnoporus sanguineus*	BRFM 1264	[39]	White-rot	Polyporales	core polyporoid clade
*Rigidoporus microporus*	ED310	[40]	White-rot	Polyporales	-
*Stereum hirsutum*	FP-91666 SS1	[27]	White-rot	Russulales	-
*Trametes pubescens*	FBCC735	[41]	White-rot	Polyporales	core polyporoid clade
*Trametes versicolor*	FP-101664 SS1	[27]	White-rot	Polyporales	core polyporoid clade
*Trametopsis cervina*	CIRM-BRFM 1824	[24]	White-rot	Polyporales	core polyporoid clade
*Amylocystis lapponica*	SKaAmylap13	[24]	Brown-rot	Polyporales	antrodia clade
*Antrodia serialis*	Sig1Antser10	[24]	Brown-rot	Polyporales	antrodia clade
*Antrodia sinuosa*	LB1	PRJNA196036	Brown-rot	Polyporales	antrodia clade
*Daedalea quercina*	L15889 ss-12	[42]	Brown-rot	Polyporales	antrodia clade
*Fibroporia radiculosa*	TFFH 294	[43]	Brown-rot	Polyporales	antrodia clade
*Fomitopsis betulina*	CIRM-BRFM 1772	[24]	Brown-rot	Polyporales	antrodia clade
*Fomitopsis pinicola*	FP-58527 SS1	[27]	Brown-rot	Polyporales	antrodia clade
*Laetiporus sulphureus*	93-53	[42]	Brown-rot	Polyporales	antrodia clade
*Postia placenta*	MAD-698-R-SB12	[44]	Brown-rot	Polyporales	antrodia clade
*Rhodofomes roseus*	CIRM-BRFM 1785	[24]	Brown-rot	Polyporales	antrodia clade
*Wolfiporia cocos*	MD-104 SS10	[27]	Brown-rot	Polyporales	antrodia clade
*Amanita muscaria*	Koide	[45]	Ectomycorrhizal	Agaricales	-
*Laccaria bicolor*	S238N-H82	[46]	Ectomycorrhizal	Agaricales	-
*Pisolithus microcarpus*	441	[45]	Ectomycorrhizal	Boletales	-
*Rhizopogon vinicolor*	AM-OR11-026	[47]	Ectomycorrhizal	Boletales	-
*Piriformospora indica*	DSM 11827	[48]	Endophyte	Sebacinales	-
*Melampsora larici-populina*	98AG31	[49]	Pathogen	Pucciniales	-
*Ustilago maydis*	521	[50]	Pathogen	Ustilaginales	-

**Table 2:** Genome features of the three newly sequenced *Ganoderma* genomes.

Genome Feature	*G. australe*	*G. leucocontextum*	*G. lingzhi*
Genome size (Mb)	84.27	60.34	60.56
Number of scaffolds	93	843	342
Scaffold L50 (bp)	1,745,385	205,166	402,014
Scaffold N50	17	66	37
Longest scaffold (bp)	4,455,856	1,715,371	2,154,085
Shortest scaffold (bp)	33,016	1008	980
GC content (%)	55.48	55.95	55.88
Protein-coding genes	20,460	16,952	15,007
Average gene length (bp)	1582	1846	1605
Complete BUSCOs (%)	84.6	99.8	73.1

## Data Availability

All data generated or analyzed during this study are included in this published article and supplementary materials. Genomic data used in this study (Table 1) are available from NCBI (https://www.ncbi.nlm.nih.gov/genome, accessed on 16 January 2022) and the JGI Genome Portal (http://genome.jgi.doe.gov, accessed on 16 January 2022). The raw sequencing data of *Ganoderma australe*, *G. lingzhi* and *G. leucocontextum* is deposited on NCBI linked to BioProject: PRJNA775667 and PRJNA791677, Biosample: SAMN22612695, SAMN22612696 and SAMN24337934, and the accession numbers are SRR16605592, SRR16605591 and JAKETP000000000.

## Supplementary Materials

The following supporting information can be downloaded at: https://www.mdpi.com/article/10.3390/jof8030311/s1, Figure S1: Synteny analyses of *Ganoderma australe* itself. The scatter plot shows correlation between all contigs, Figure S2: Macrosynteny between *Ganoderma* species. The percentage of syntenic hits occurring in the same order on the compared block of sequences between two genomes are shown inside each Circos plot, Table S1: Model list for each orthologous group of concatenate protein alignments, Table S2: Main features of the 58 genomes used in this study, Table S3: (A) Size and genome coverage of the 10 largest scaffolds in *Ganoderma* genomes, (B) synteny summary statistic of the 10 largest scaffolds in *Ganoderma* species, Table S4: Summary statistics of components and coverage for transposable elements (TEs) in the 58 genomes, Table S5: Number of the gene clusters involved in secondary metabolites biosynthesis identified in the 58 genomes, Table S6: Number of the filtered protein domains (abundance > 100) in Pfam database among the 58 genomes, Table S7: Count of each category (CAZymes, lipases, SSP, proteases) in 58 fungal secretomes, Table S8: Number of small secreted proteins in 58 secretomes, Table S9: Number of secreted CAZymes in 58 secretomes, Table S10: Composition and ratios of secreted CAZymes for plant and microbial cell wall degradation in 58 genomes, Table S11: Number of secreted proteases in 58 secretomes, Table S12: Number of secreted lipases in 58 secretomes.
