# Supplementary figures and images for: Phylogenomics and Comparative Genomics Highlight Specific Genetic Features in Ganoderma Species
Source: J Fungi (Basel). 2022 Mar 18;8(3):311. doi: 10.3390/jof8030311 (PMC8955403; doi:10.3390/jof8030311)

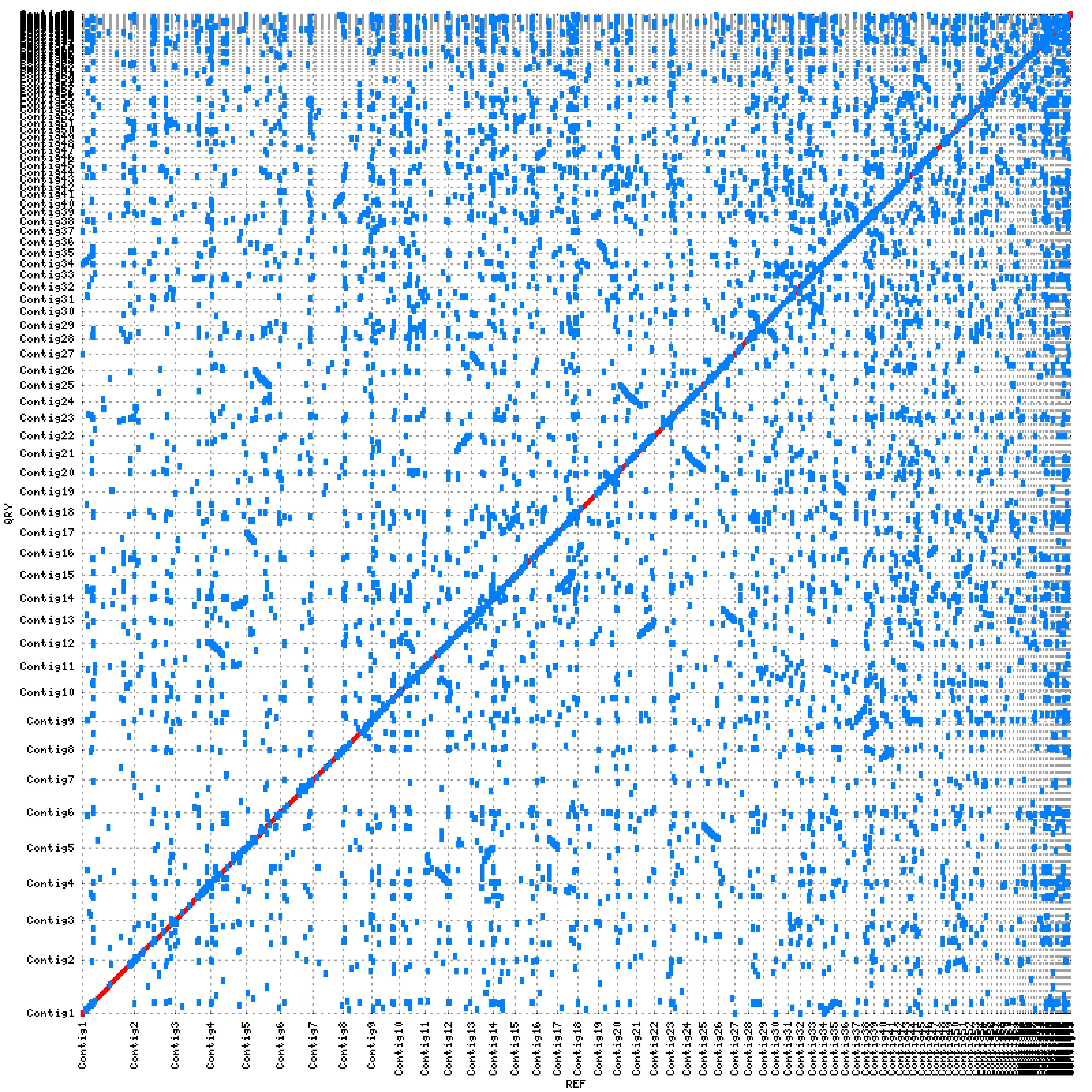

Supplement: Supplementary file 1 [file jof-08-00311-s001.zip › Figure S1.jpg]

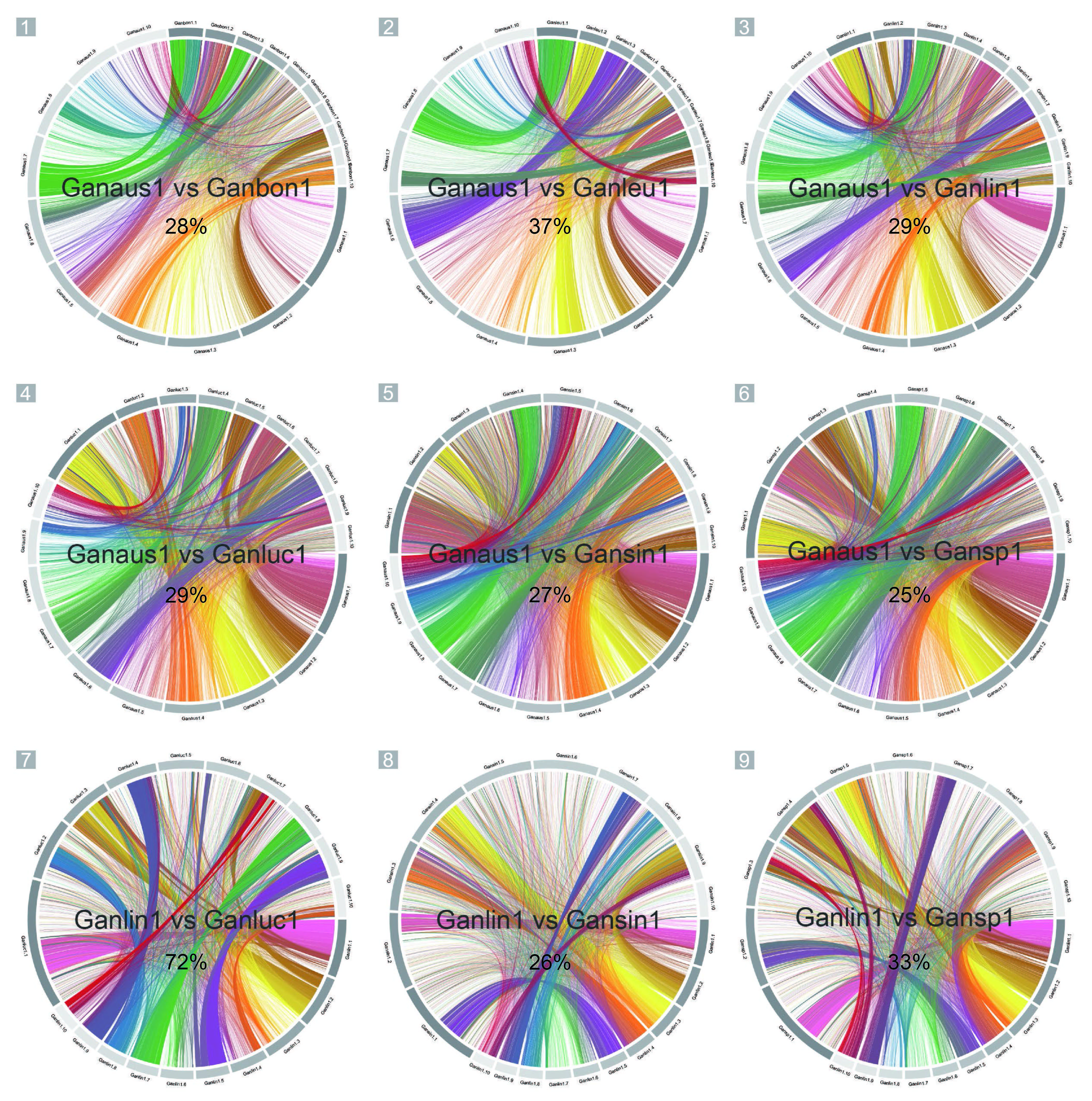

Supplement: Supplementary file 1 [file jof-08-00311-s001.zip › Figure S2-1.jpg]

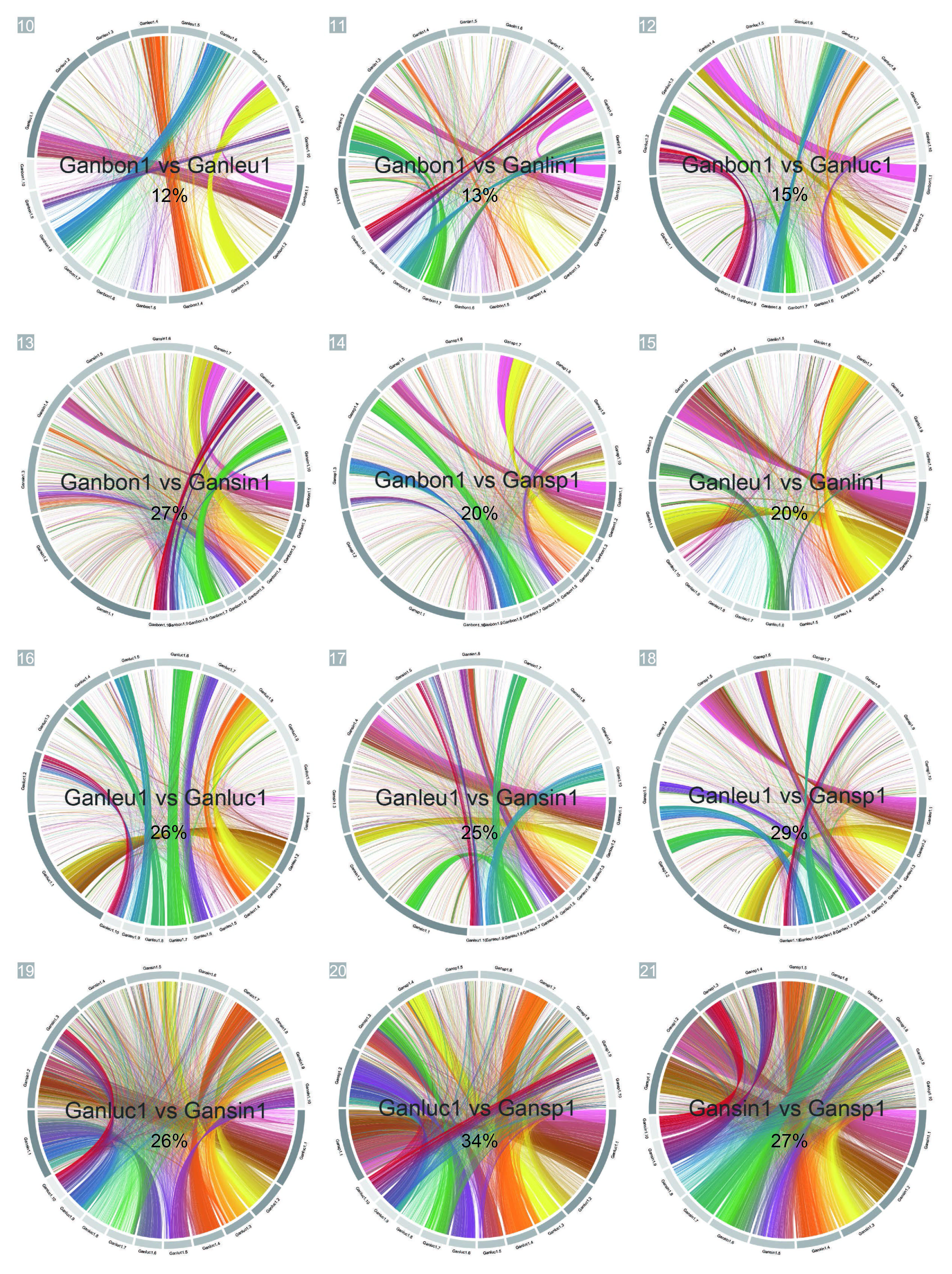

Supplement: Supplementary file 1 [file jof-08-00311-s001.zip › Figure S2-2.jpg]
